# Association of Adherence to the Mediterranean-Style Diet with Lower Frailty Index in Older Adults

**DOI:** 10.3390/nu13041129

**Published:** 2021-03-30

**Authors:** Toshiko Tanaka, Sameera A. Talegawkar, Yichen Jin, Stephania Bandinelli, Luigi Ferrucci

**Affiliations:** 1 Longitudinal Study Section, Translation Gerontology Branch, National Institute on Aging, Baltimore, MD 21224, USA; FerrucciLu@grc.nia.nih.gov; 2 Department of Exercise and Nutrition Sciences, Milken Institute School of Public Health, The George Washington University, Washington, DC 20052, USA; stalega1@gwu.edu (S.A.T.); yjin@gwu.edu (Y.J.); 3 Geriatric Unit, Azienda Sanitaria Toscana Centro, 50125 Firenze, Italy; stefania1.bandinelli@uslcentro.toscana.it

**Keywords:** Mediterranean diet, frailty index, trajectory

## Abstract

Identifying modifying protective factors to promote healthy aging is of utmost public health importance. The frailty index (FI) reflects the accumulation of health deficits and is one widely used method to assess health trajectories in aging. Adherence to a Mediterranean-type diet (MTD) has been associated with favorable health trajectories. Therefore, this study explored whether adherence to a MTD is negatively associated with FI in the InCHIANTI study. Participants (*n* = 485) included individuals over 65 years of age at baseline with complete data over a follow-up period of 10 years. MTD was computed on a scale of 0–9 and categorized based on these scores into three groups of low (≤3), medium (4–5), and high (≥6) adherence. Being in a high or medium adherence group was associated with 0.03 and 0.013 unit lower FI scores over the follow-up period, compared to the low adherence group. In participants with a low FI at baseline, being in a high or medium MTD-adherence group had 0.004 and 0.005 unit/year slower progression of FI compared to the low adherence group. These study results support adherence to a MTD as a protective strategy to maintain a lower FI.

## 1. Introduction

As lifespan extends globally, there is a greater emphasis on improving health span. There are various metrics to evaluate “health” during the lifespan. It is generally recognized that the development of frailty is an important turning point in the trajectory of health in older persons. The frailty index (FI), a cumulative score of health deficits, is one of the most frequently used operational definitions of frailty [1,2]. The FI is calculated as a proportion of health deficits based on a varying (<30–70) number of variables reflecting symptoms, signs, diseases, and disabilities that accumulate over time. Although the variables included in the FI can vary from study to study, there is a strong rationale that they capture an important dimension of health in old age.

In the InCHIANTI study, an Italian prospective cohort study, the FI was operationalized using 42 variables that reflected age-related disease diagnosis, physical function, and cognitive health [3]. The FI at baseline was predictive of all-cause and cardiovascular disease mortality [3]. Similar associations between the FI and aging outcomes including major mobility disability [4], cardiovascular disease mortality [5], and all-cause mortality [6] have been reported in other cohort studies. These results show that the FI is an important indicator of health-span and identifying factors, particularly modifiable factors, that can improve FI is important.

Diet is one of the most important modifiable risk factors for various age-related conditions. A Mediterranean-type diet (MTD) that is characterized by higher daily intake of plant-based foods (vegetables, fruits, nuts, legumes, and cereals), fish, and monosaturated fats (primarily from olive oil), and lower intake of meats and saturated fats, with moderate intake of alcohol, has been associated with various aging conditions including cognitive decline [7], physical function [8,9], multimorbidity [10], and mortality [11]. We have previously shown that adherence to a MTD was associated with lower risk for the development of the frailty phenotype [12]. The frailty phenotype is another commonly studied frailty construct based on five criteria (unintentional weight loss, low grip strength, low energy, low walking speed, and low physical activity) that complements but is not identical to the FI. Thus, in this study, we examined the hypothesis that adherence to a MTD is associated with a lower FI in participants in the InCHIANTI study.

## 2. Materials and Methods

### 2.1. Study Population

The InCHIANTI study is a prospective cohort of older subjects living in the Chianti region in Tuscany, Italy. The primary aims of the study are to understand the factors contributing to mobility disability in aging. The details of the study have been described in detail [13]; in brief, subjects between 21 and 102 years of age (*n* = 1453) were recruited from the population registry of Greve in Chianti and Bagno a Ripoli at a participation rate of 90%. Participants were followed every three years from the baseline visit (1998–2000) to three follow-up visits. In the analysis, we included 825 participants over 65 at baseline with at least one follow-up visit. The study protocol was approved by the Italian National Institute of Research and, in the United States, the protocol was given an exemption status by the Office of Human Subject Research Protection (Exemption #11976).

### 2.2. Dietary Assessment and Mediterranean Diet Score Construction

At the baseline visit, dietary intake in the past year was assessed using a food frequency questionnaire (FFQ) adapted from the European Prospective Investigation on Cancer and Nutrition study, which was validated for use in the InCHIANTI study [14]. The Mediterranean-type diet score (MTD) was constructed using the algorithm developed by Trichopoulou et al. [15] as previously described [12]. Briefly, consumption of nine food groups was dichotomized using sex-specific median consumption as cutoffs. For “beneficial” foods (vegetables, legumes, fruits, cereal, fish, and a ratio of monounsaturated fats(MUFA):saturated fats(SFA)), consumption above the median was assigned a score of 1 and below the median was assigned a score of 0. Conversely, for detrimental foods (meat and dairy products), a score of 1 was assigned for consumption under the median and a score of 0 for consumption above the median. The median scores in men and women for each food group were as follows: 148.1 and 134.1 g (vegetables), 16 and 13.9 g (legumes), 273 and 261.8 g (fruits and nuts), 281.7 and 206.4 g (cereal), 23.6 and 20.4 g (fish), 1.5 and 1.4 (ratio of MUFA:SFA), 115.3 and 94.2 g (meat), and 153.4 and 173.6 g (dairy). For alcohol, a score of 1 was assigned to those who consumed between 10 and 50 g/d or 5 and 25 g/d in men and women respectively. Total MTD was derived as a sum of these scores and ranged from 0 (low adherence to a MTD) to 9 (maximal adherence). For analysis, the score was categorized into three groups as follows: low adherence (MTD ≤ 3), medium adherence (MTD 4–5), and high adherence (MTD ≥ 6). For all analyses, the low adherence group was used as a reference group.

### 2.3. Assessment of Frailty Index (FI)

The variable selection and operationalization of the FI in the InCHIANTI study were previously described [3]. In brief, 42 variables that represent health deficits and different functional domains were selected. These variables included major chronic medical conditions (hypertension, myocardial infarction, congestive heart failure, chronic liver disease, cancer, peripheral arterial disease, stroke, Parkinson’s disease, diabetes, chronic lung disease, angina pectoris, and knee/hip arthritis) [16], difficulties with various activities of daily living (ADL) and instrumental ADL (IADL) [17,18], self-rated health, depressive symptoms (Center for Epidemiologic Studies Depression (CES-D) [19], subdomains of the Mini-Mental State Examination (MMSE) [20], self-reported weight loss, physical activity level in the past year, gait speed, and grip strength. An FI was calculated in participants with less than 20% of these 42 variables missing (*n* = 12). The FI was calculated as a ratio of the sum of all components to the total number of non-missing components and ranged from 0 to 1, reflecting having no deficits to having all deficits. 

### 2.4. Measurement of Main Covariates

Clinical and demographic factors that were associated with the FI in univariate analyses were considered as covariates in the final analyses. Sociodemographic information such as age, sex, and years of education, was collected during a structured interview. Self-reported smoking status was categorized into three groups of never smokers, former smokers, or current smokers (within 3 years). Physical activity level in the past year was assessed using an interviewer-administered questionnaire [21]. A body mass index (BMI) was calculated as weight (kg) divided by squared height (meter). Plasma C-reactive protein (CRP) was measured using colorimetric competitive immunoassay (Roche Diagnostics, GmbH, Mannheim, Germany) and plasma IL-6 was measured using Bio-source cytoscreen ultrasensitivity kits. Plasma fatty acids omega-3 and omega-6 were measured using gas chromatography as previously described [22]. Plasma carotenoids, tocopherols, and MUFA were measured via high-performance liquid chromatography (HPLC) [23,24] and gas chromatography [22], respectively.

### 2.5. Statistical Analysis

Differences in baseline characteristics were tested using an analysis of variance for continuous variable and a chi-square for categorical variables. A cross-sectional analysis of adherence to the MTD and FI was assessed using linear regression using lm() function in R (version 3.6.2). An analysis of the longitudinal trajectories of FI was assessed using a linear mixed model with lmer() function from the lme4 package, using follow-up time as the time metric. Differences in trajectory (or slope) of the FI by MTD group were tested using an interaction term between the MTD group and follow-up time. For both cross-sectional and longitudinal analysis, the models were adjusted for age (at baseline), sex, study site (Greve or Bagno a Ripoli), total energy intake, smoking status, IL-6, CRP, BMI, years of education, and plasma levels of α-tocopherol, β-carotene, α-carotene, and monosaturated fatty acids (MUFA). To test whether association of MTD with FI trajectory differed by baseline FI, a stratified analysis was conducted in a sample split by the median baseline FI value. For all analyses, statistical significance was considered at *p* ≤ 0.05. All analyses were conducted using R version 3.6.2.

## 3. Results

### 3.1. Association of Mediterranean-Type Diet with FI at Baseline

**Figure 1:**
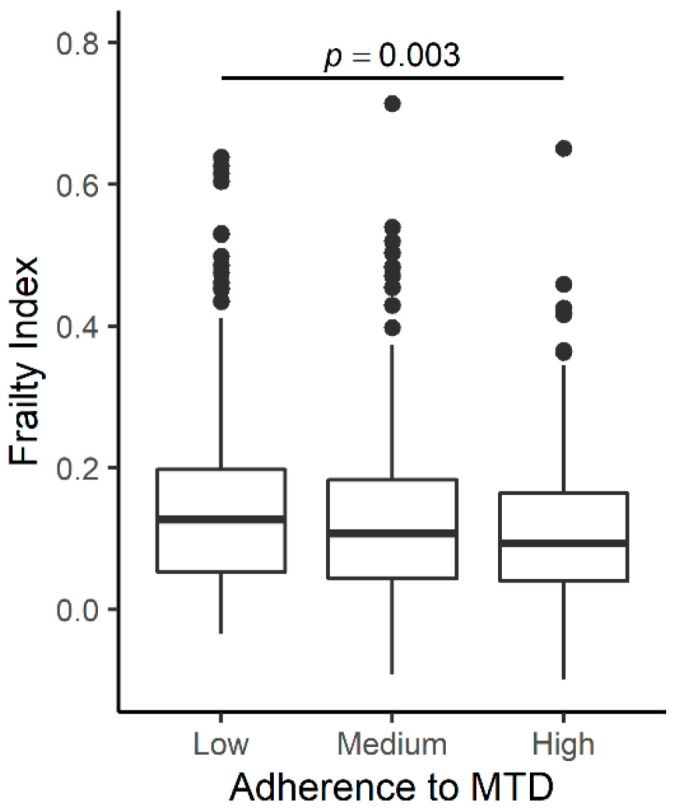
Association of adherence to a Mediterranean-type diet (MTD) at baseline. The boxplot displays the mean value of frailty index (FI) for subjects with low (MTD ≤ 3), medium (MTD 4–5), and high (MTD ≥ 6) adherence to a MTD. Significant differences were observed between low and high adherence group (*p* = 0.0322).

Compared to participants with follow-up data used in this analysis (*n* = 825), those who did not have follow-up data (*n* = 180) had profiles consistent with poorer health, including higher FI; older age; fewer years of education; lower MMSE; lower total energy intake; higher plasma concentrations of IL-6, CRP, and monounsaturated fatty acid (MUFA); and lower plasma concentrations of α-tocopherol, α-carotene, and β-carotene (Supplemental Table S1). At the baseline visit, the average age of participants with follow-up data was 73.5 years, and 56.1% were women (Table 1). FI ranged from 0.01 to 0.69 with a median value of 0.103. Participants with medium or high adherence to MTD were younger, had lower plasma CRP concentrations, greater total energy intake, and higher plasma b-carotene concentrations compared to the low adherence group. The highest percentage of women was in the medium adherence group, and the lowest percentage was found in the highest adherence group. No differences by MTD group were found for the study site; smoking status; BMI; years of education; MMSE score; and plasma concentrations of IL-6, α-tocopherol, and monosaturated fatty acids (MUFA). Those in the high adherence group had the lowest FI score, followed by the medium and low adherence group. When association was adjusted for covariates (age; sex; study site; total energy intake; years of education; smoking status; BMI; and plasma concentrations of MUFA, α-carotene, β-carotene, α-tocopherol, CRP, and IL-6) the association remained, with significant differences between the low and high adherence group (Figure 1).

To investigate which component had the strongest association with the FI, individual food groups were analyzed. In the unadjusted model, the consumption of vegetables and alcohol was significantly associated with the FI (Supplemental Table S2). In the fully adjusted model, alcohol consumption remained significantly inversely associated with the FI.

### 3.2. Association of the Mediterranean-Type Diet with the Trajectory of FI

Participants were followed for a mean of 7.35 years (range from 1.9 to 10 years), and their FI increased an average of 0.014 units per year. Older baseline age; higher BMI; smokers; women; and higher plasma concentrations of CRP, MUFA, and α-carotene were associated with a higher FI across the whole follow-up period (Supplemental Table S3).

**Figure 2:**
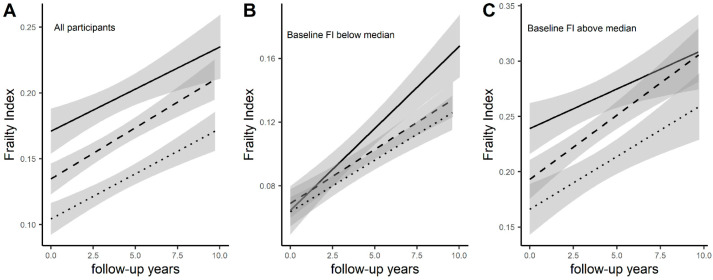
Association of MTD with trajectories of the Mediterranean-type diet (MTD). The associations of the MTD and the trajectory of frailty index (FI) were tested using a linear mixed model in all participants (**A**), participants with baseline FI below (**B**) or above (**C**) the median value of 0.103. The trajectories are stratified by low (solid line), medium (dashed line), or high (dotted line) adherence to a MTD. The trajectory of FI differed by MTD in subjects with low FI at baseline where the low adherence group had a faster progression of FI over time compared with the medium and high adherence groups.

Conversely, higher β-carotene, vitamin E, and total energy intake were associated with a lower FI during the follow-up period. Compared to the low MTD-adherence group, the high adherence group had 0.03 units lower FI (*p* < 0.0001) and the medium adherence group had 0.013 units lower FI (*p* = 0.0164). The associations between adherence to the MTD and the FI remained significant after adjustment of covariates, and there was little change in the effect sizes (Supplemental Table S3). The high adherence group had 0.006 units slower progression of FI compared to the low adherence group (Table 2). No differences in the trajectory of the FI were observed for the medium adherence group (Table 2, Figure 2A). 

Analyses of each MTD component show that alcohol consumption is associated with a lower FI throughout the follow-up period (Supplemental Table S4). There was signification interaction between the consumption of vegetables and fish with time indicating a slower trajectory of FI (Supplemental Table S4). 

### 3.3. Association of the Mediterranean-Type Diet with the Trajectory of FI by Baseline FI

We tested whether the association of the MTD with the FI differed by baseline FI values. In participants with a low FI (below the median value), there were significant differences in the slope of FI incline over time (Table 2). Compared to the low adherence to MTD group, both the medium and high adherence group had 0.004 and 0.005 unit/year slower progression in FI, respectively (Table 2, Figure 2B). In participants with a high FI (above the median value), there were no differences in the trajectory of FI increase and those in the higher MTD-adherence group maintained a lower FI throughout the follow-up period (Figure 2C).

## 4. Discussion

In this study, we report that adherence to the MTD is associated with a better FI over a 10 year follow-up period. We observed that the effect of the MTD on trajectories of FI depended on the participant’s FI status at baseline. Overall, adherence to the MTD is protective against the progression of the FI, however, in participants that have a low FI at baseline low adherence to a MTD displayed faster worsening of the FI. This suggests that, for people who are more robust from the standpoint of the FI, promoting greater adherence to the MTD may be more beneficial in preventing health decline. All associations observed were independent of other risk factors, including inflammation, smoking status, years of education, and plasma nutrient biomarkers (vitamin E, carotenoids, and MUFA) at baseline. 

There have been many studies that have examined the relationship between adherence to the MTD and the frailty phenotype. While the frailty phenotype and FI are correlated traits and often discussed interchangeably, the two traits are in fact complementary and independent constructs [25,26]. While the variables included in the FI are not predefined, the frailty phenotype is a construct from five defined criteria: unintentional weight loss, low grip strength, low energy, low walking speed, and low physical activity [27]. Frailty is considered when three or more of these conditions are met. Unlike the FI that is a continuous variable, the frailty phenotype is a categorical variable. There have been many studies that have shown the protective effect of adherence to a MTD on the incidence of frailty defined using the frailty phenotype [28,29,30]. A meta-analysis of four representative longitudinal studies of a MTD and the frailty phenotype showed that, over an average follow-up period of 3.9 years, medium and high adherence to a MTD was associated with 40% and 60% lower odds of developing frailty, respectively [31]. These studies reflect the overwhelming support for using a MTD in the prevention of frailty. The relative ease of operationalizing the frailty phenotype using the five set criteria perhaps explains why the number of studies examining the frailty phenotype with a MTD outweighs studies examining the FI. However, it is important to understand the effect the MTD exhibits on both frailty constructs since the specific variables required to ascertain the frailty phenotype are not available in many studies. 

The associations of a MTD and three frailty constructs, including the frailty phenotype, the 61-variable FI, and the Tilburg Frailty Indicator, were investigated in 1740 subjects over 65 years of age in the Hellenic Longitudinal Investigation of Aging and Diet (HELIAD) study [30]. Higher adherence to the MTD was associated with a 4% decrease in the odds of frailty based on the 61-variable FI. There was also a trend for lower odds of frailty based on frailty phenotype, but the association was not significant. Interestingly, in this population the prevalence of frailty based on the three definitions differed considerably (4%–frailty phenotype, 18.7%–61-variable FI, and 25.4%–Tilburg Frailty Indicator) confirming the notion that these frailty constructs are different. The results from the InCHIANTI study are consistent with this prior report. Moreover we show that adherence to a MTD has the beneficial effect of maintaining a lower FI over a 10 year follow-up period. Moreover, our study indicates that low adherence to a MTD may accelerate the increase in the FI over time in those who have a low FI at baseline. The longitudinal effect of diet on the FI has been shown in another study, where the association of dietary patterns with changes in the FI over time was examined in 2632 individuals from the Rotterdam study [32]. Adherence to the Dutch national dietary guidelines was found to be associated with a lower FI both at baseline and over a 4 year follow-up period. Using principal component analysis, three dietary patterns were developed, including a “Traditional” pattern, characterized by higher intake of legumes, eggs, and savory snacks; a “Carnivore” pattern, with higher intake of meat and poultry; and a “Health Conscious” pattern, with higher intake of whole grain products, vegetables, and fruit. Interestingly, none of these patterns were associated with the FI at baseline but the “Traditional” pattern was associated with less frailty over time. This study, along with our study, suggests that following a higher quality diet can have long-lasting effects on the FI.

Adherence to a MTD is thought to promote health through various mechanisms. A MTD emphasizes the consumption of nutrient-rich foods, and as such, adherence to a MTD is associated with better nutrient status, including plasma levels of carotenoids and fatty acids [33,34,35]. Following a MTD is associated with a favorable chronic disease risk profile such as reduced blood cholesterol, lower inflammation, and increased antioxidant capacity [33,36,37,38]. Thus, adherence to a MTD most likely confers protection from the FI through these multiple mechanisms. In our study, the association of a MTD with the FI was independent of several of these factors, including nutrient status (carotenoids, vitamin E, and MUFA) and inflammation (CRP and IL-6), suggesting that the beneficial effects of a MTD cannot be fully explained by these factors. 

This study has several strengths. First, this study was conducted in a well-characterized study that included repeated measures of the FI over a 10 year period, thereby allowing us to evaluate the long-term effect of the baseline diet. The study has comprehensive data on each participant, enabling us to adjust for several variables that may be important confounders or covariates in the relationship between the MTD and the FI. The study has several limitations. While the InCHIANTI study’s data are rich, there likely are confounding factors that were not measured in our study. The MTD score was measured by self-report using a FFQ that is known to introduce some measurement error [39].

## 5. Conclusions

In conclusion, this study provides evidence that following a MTD has protective association with the FI. In particular, adherence to a MTD may be particularly important in older individuals who have low FI or are robust to maintain their health status. As the aging population grows worldwide, ensuring the health of this subgroup is of utmost importance. Our data suggest that promoting adherence to a MTD could be an effective strategy to reduce the burden of health deficits in older individuals.

## Figures and Tables

**Table 1:** Baseline demographic and clinical characteristics of InCHIANTI participants by adherence to the Mediterranean-type diet.

	All		Low Adherence		Medium Adherence		High Adherence		
	Mean/*n*	(SD/%)	Mean/*n*	(SD/%)	Mean/*n*	(SD/%)	Mean/*n*	(SD/%)	*p* *
*n*	825		235		357		233		
Age (y)	73.5	(6.4)	75.1	(7.0)	73.6	(6.5)	71.9	(5.2)	<0.001
Sex (%Female)	463	(56.1)	131	(55.7)	219	(61.3)	113	(48.5)	0.009
Site (%Bagno a Ripoli)	441	(53.5)	112	(47.7)	195	(54.6)	134	(57.5)	0.086
Smoking (%Smoker)	118	(14.3)	36	(15.3)	46	(12.9)	36	(15.5)	0.596
IL6 (pg/mL)	2.05	(3.39)	2.09	(2.24)	1.90	(1.96)	2.23	(5.47)	0.441
CRP (ug/mL)	4.59	(7.39)	5.40	(10.73)	3.89	(4.45)	4.87	(6.83)	0.035
BMI (kg/m^2^)	27.5	(4.0)	27.1	(4.2)	27.5	(4.2)	27.8	(3.6)	0.128
Years of Education (y)	5.58	(3.31)	5.53	(3.67)	5.45	(3.10)	5.85	(3.23)	0.311
MMSE	25.4	(3.4)	25.1	(3.8)	25.4	(3.3)	25.8	(3.0)	0.063
Total energy intake (kcal/day)	1942.7	(566.1)	1826.3	(582.1)	1917.9	(571.8)	2098.1	(505.7)	<0.001
Plasma α-tocopherol (µmol/L)	30.4	(8.3)	29.4	(8.3)	30.9	(8.3)	30.8	(8.2)	0.081
Plasma α-carotene (µmol/L)	0.06	(0.06)	0.05	(0.04)	0.06	(0.04)	0.07	(0.08)	0.144
Plasma β-carotene (µmol/L)	0.43	(0.26)	0.39	(0.24)	0.45	(0.28)	0.44	(0.25)	0.021
Plasma monosaturated fatty acid *	33.0	(3.7)	32.5	(3.8)	33.0	(3.7)	33.4	(3.8)	0.055
Frailty Index	0.13	(0.10)	0.16	(0.12)	0.13	(0.09)	0.11	(0.08)	<0.001

* *p*-values from one-way ANOVA or chi-square test.

**Table 2:** Association of adherence to a Mediterranean-type diet at baseline with trajectories of the frailty index over 10 years.

	Model without Interaction			Model with Interaction		
**All participants**						
Adherence to MTD	**Beta**	**SE**	***p***	**Beta**	**SE**	***p***
Low	Reference			Reference		
Medium	−0.013	0.005	0.016	−0.010	0.006	0.099
High	−0.030	0.006	<0.001	−0.023	0.007	<0.001
Follow-up time	0.013	0.001	<0.001	0.016	0.002	<0.001
Low × Follow-up time				Reference		
Medium × Follow-up time				−0.003	0.002	0.249
High × Follow-up time				−0.006	0.002	0.021
**Low frailty index at baseline**						
Adherence to MTD	**Beta**	**SE**	***p***	**Beta**	**SE**	***p***
Low	Reference			Reference		
Medium	−0.001	0.004	0.820	0.002	0.005	0.612
High	−0.006	0.005	0.164	−0.003	0.005	0.574
Follow-up time	0.009	0.001	<0.001	0.012	0.002	<0.001
Low × Follow-up time				Reference		
Medium × Follow-up time				−0.004	0.002	0.040
High × Follow-up time				−0.005	0.002	0.030
**High frailty index at baseline**						
Adherence to MTD	**Beta**	**SE**	***p***	**Beta**	**SE**	***p***
Low	Reference			Reference		
Medium	−0.010	0.009	0.248	−0.010	0.010	0.295
High	−0.021	0.011	0.057	−0.016	0.012	0.186
Follow-up time	0.017	0.001	<0.001	0.018	0.003	<0.001
Low × Follow-up time				Reference		
Medium × Follow-up time				0.000	0.004	0.959
High × Follow-up time				−0.004	0.004	0.363

## Data Availability

InCHIANTI data is available through submission of research proposal at inchiantistudy.net.

## Supplementary Materials

The following are available online at https://www.mdpi.com/article/10.3390/nu13041129/s1, Table S1. Comparison of demographic and clinical characteristics of InCHIANTI participants with and without follow up data; Table S2. Association of adherence to components Mediterranean-type diet with frailty index at baseline visit; Table S3. Association of adherence to Mediterranean-type diet at baseline with trajectories of frailty index over 10 years; Table S4. Association of components of Mediterranean-type diet at baseline with trajectories of frailty index over 10 years.
