# Supplementary material for: Association of Adherence to the Mediterranean-Style Diet with Lower Frailty Index in Older Adults
Source: Nutrients. 2021 Mar 30;13(4):1129. doi: 10.3390/nu13041129 (PMC8065455; doi:10.3390/nu13041129)
Supplement: Supplementary file 1 [file nutrients-13-01129-s001.pdf]

nutrients

Table S1. Comparison of demographic and clinical characteristics of InCHIANTI participants with and without follow up data.

	No follow up data		With Follow up data		
	Mean/*n*	(SD/%)	Mean/*n*	(SD/%)	*p*
n	180		825		
Age (yrs)	80.0	(8.0)	73.5	(6.4)	<0.001
Sex (%Female)	98	(54.4)	463	(56.1)	0.743
Site (%Bagno a Ripoli)	81	(45.0)	441	(53.5)	0.048
Smoking (%Smoker)	18	(10.0)	118	(14.3)	0.159
IL6 (pg/mL)	3.22	(7.12)	2.05	(3.39)	0.033
CRP (ug/mL)	8.46	(15.91)	4.59	(7.39)	0.002
BMI (kg/m^2)	27.3	(4.4)	27.5	(4.0)	0.647
Years of Education (years)	4.42	(2.87)	5.58	(3.31)	<0.001
MMSE	21.4	(6.9)	25.4	(3.4)	<0.001
Total energy intake (kcal/day)	1811.6	(519.4)	1942.7	(566.1)	0.003
α-tocopherol (µmol/L)	28.5	(8.9)	30.4	(8.3)	0.009
α-carotene (µmol/L)	0.05	(0.04)	0.06	(0.06)	0.035
β-carotene (µmol/L)	0.37	(0.23)	0.43	(0.26)	0.004
Plasma monosaturated fatty acid	34.1	(3.8)	33.0	(3.7)	0.001
Frailty Index	0.26	(0.19)	0.13	(0.10)	<0.001

Table S2. Association of adherence to components Mediterranean-type diet with frailty index at baseline visit.

	Base Model			Fully Adjusted Model		
	Beta	SE	*p*	b	SE	*p*
Vegetables	-0.017	0.006	0.0086	-0.011	0.006	0.0746
Legumes	-0.008	0.006	0.1934	-0.003	0.006	0.5967
Fruits and nuts	-0.012	0.006	0.0586	-0.011	0.006	0.0838
Cereal	0.009	0.008	0.2158	0.001	0.007	0.9001
Fish	-0.003	0.006	0.5987	-0.006	0.006	0.2717
MUFA:SFA	-0.011	0.006	0.0672	-0.008	0.006	0.1663
Meat	-0.005	0.006	0.4635	-0.003	0.006	0.6430
Dairy	0.002	0.006	0.7933	-0.001	0.006	0.9064
Alcohol	-0.018	0.006	0.0036	-0.014	0.006	0.0236

Table S3. Association of adherence to Mediterranean-type diet at baseline with trajectories of frailty index over 10 years.

	Base Model			Fully Adjusted Model		
	Beta	SE	*p*	Beta	SE	*p*
Adherence to MTD						
Low		Reference			Reference	
Medium	-0.020	0.005	0.0002	-0.013	0.005	0.0164
High	-0.030	0.006	<0.0001	-0.030	0.006	<0.0001
Follow-up time	0.013	0.001	<0.0001	0.013	0.001	<0.0001
Baseline Age	0.008	0.000	<0.0001	0.007	0.000	<0.0001
Sex (women)	0.026	0.005	<0.0001	0.035	0.005	<0.0001
Site (Bagno a Ripoli)	-0.012	0.004	0.0050	-0.005	0.005	0.2672
Total Energy Intake	-0.009	0.003	0.0006	-0.005	0.003	0.0572
Smoking				0.011	0.007	0.1020
IL-6				0.002	0.001	<0.0001
CRP				-0.002	0.001	0.0095
BMI				0.001	0.001	0.2871
Years of Education				0.001	0.000	<0.0001
Plasma α-tocopherol				-0.001	0.000	0.0031
Plasma α-carotene				0.105	0.047	0.0239
Plasma β-carotene				-0.039	0.010	<0.0001
Plasma monosaturated fatty acid				0.002	0.001	0.0063

Table S4. Association of components of Mediterranean-type diet at baseline with trajectories of frailty index over 10 years.

	Main effect Diet[1]			Main effect Time[1]			Interaction (Dietxtime)[1]		
	Beta	SE	*p*	Beta	SE	*p*	Beta	SE	*p*
Vegetable	-0.004	0.005	0.4360	0.016	0.001	1.95E-32	-0.006	0.002	0.0006
Legumes	-0.006	0.005	0.2627	0.014	0.001	9.67E-25	-0.002	0.002	0.2743
Fruit	-0.010	0.005	0.0514	0.014	0.001	3.29E-22	-0.001	0.002	0.5444
Cereal	0.002	0.006	0.7537	0.013	0.001	1.51E-23	-0.001	0.002	0.5951
Fish	-0.005	0.005	0.3285	0.015	0.001	1.01E-27	-0.004	0.002	0.0417
MUFA:SFA	-0.006	0.005	0.2747	0.014	0.001	4.68E-25	-0.003	0.002	0.1226
Meat	-0.008	0.005	0.1377	0.012	0.001	4.81E-18	0.002	0.002	0.1867
Dairy	-0.003	0.005	0.5376	0.013	0.001	5.34E-23	-0.001	0.002	0.7201
Alcohol	-0.014	0.005	0.0052	0.013	0.001	1.75E-25	0.0002	0.002	0.9080

[1]Results from linear mixed effects model.
